# Correlation between stem cell molecular phenotype and atherosclerotic plaque neointima formation and analysis of stem cell signal pathways

**DOI:** 10.3389/fcell.2023.1080563

**Published:** 2023-01-12

**Authors:** Chuanxin Shi, Kefan Zhang, Zhenyu Zhao, Yifan Wang, Haozhe Xu, Wei Wei

**Affiliations:** ^1^ Division of General Surgery, The Second Affiliated Hospital of Nanjing Medical University, Nanjing, China; ^2^ Department of Biotherapy, Medical Center for Digestive Diseases, The Second Affiliated Hospital of Nanjing Medical University, Nanjing, China

**Keywords:** stem cells, stem cell markers, molecular phenotype, neointima, atherosclerotic disease, signaling pathways

## Abstract

Vascular stem cells exist in the three-layer structure of blood vessel walls and play an indispensable role in angiogenesis under physiological conditions and vascular remodeling under pathological conditions. Vascular stem cells are mostly quiescent, but can be activated in response to injury and participate in endothelial repair and neointima formation. Extensive studies have demonstrated the differentiation potential of stem/progenitor cells to repair endothelium and participate in neointima formation during vascular remodeling. The stem cell population has markers on the surface of the cells that can be used to identify this cell population. The main positive markers include Stem cell antigen-1 (Sca1), Sry-box transcription factor 10 (SOX10). Stromal cell antigen 1 (Stro-1) and Stem cell growth factor receptor kit (c-kit) are still controversial. Different parts of the vessel have different stem cell populations and multiple markers. In this review, we trace the role of vascular stem/progenitor cells in the progression of atherosclerosis and neointima formation, focusing on the expression of stem cell molecular markers that occur during neointima formation and vascular repair, as well as the molecular phenotypic changes that occur during differentiation of different stem cell types. To explore the correlation between stem cell molecular markers and atherosclerotic diseases and neointima formation, summarize the differential changes of molecular phenotype during the differentiation of stem cells into smooth muscle cells and endothelial cells, and further analyze the signaling pathways and molecular mechanisms of stem cells expressing different positive markers participating in intima formation and vascular repair. Summarizing the limitations of stem cells in the prevention and treatment of atherosclerotic diseases and the pressing issues that need to be addressed, we provide a feasible scheme for studying the signaling pathways of vascular stem cells involved in vascular diseases.

## 1 Introduction

Atherosclerosis is the formation of fibrofatty lesions in the arterial wall (Libby et al., 2019). It is mainly characterized by endothelial cell dyskinesia, inflammatory cell recruitment and vascular smooth muscle cell dedifferentiation (Gimbrone and García-Cardeña, 2016; Wang et al., 2018). Review the evolution of atherosclerosis, which affects younger adults more than ever and is involved in the majority of deaths. As a systemic disease, its main affected sites include cardiovascular, cerebrovascular and peripheral vessels (Poznyak et al., 2020). When present, the disease typically manifests as most myocardial infarctions and numerous strokes, as well as disabling peripheral arterial disease. The occurrence and development of atherosclerosis is a complex process, and the current exploration of the pathogenesis of atherosclerosis highlights the influence of mutations in bone marrow and stem cells on the risk of cardiovascular disease (Libby, 2021). The influence of related pathways on inflammation may further modulate the progression of atherosclerosis (Zhu et al., 2018). Currently, various experimental data indicate that atherosclerosis is a chronic, immune, inflammatory vascular disease driven primarily by innate immune responses of myeloid cells (Ling et al., 2021). Chronic vascular inflammation can attract cells of the innate and adaptive immune systems to lesions, thereby inhibiting or accelerating atherosclerosis (Kobiyama and Ley, 2018; Saigusa et al., 2020). Traditionally, atherosclerosis has been considered a cholesterol storage disease. The accumulation of low-density lipoprotein triggers vascular inflammation. Macrophages absorb lipoprotein through micropinocytosis or aggregate in the form of cholesterol crystals. As cholesterol continues to flow in, macrophages gradually transform into foam cells (Wolf and Ley, 2019). Compelling experimental and clinical data suggest that inflammation is fundamentally involved in the pathophysiology of atherogenesis (Libby and Hansson, 2019). Inflammation may serve as a link between various factors and atherosclerosis, but it does not replace lipid risk (Libby, 2021). Inflammation is associated with infiltration of various immune cells, most notably macrophages, T cells (Williams et al., 2020). Stem cells are cells with the potential for self-renewal and differentiation, and a key role in the pathogenesis of atherosclerosis has been demonstrated (Orlandi, 2015). Inflammatory pathways in stem cells are regulated by multiple transcription factors and co-regulatory molecules (Rahman et al., 2016). However, the signaling pathways that regulate inflammation and cytokine production are still not completely understood. Atherosclerosis is a multifactorial disease. As the disease progresses, it is gradual and hidden, and for a long time there is no apparent discomfort. It is hard to get noticed. Once it occurs, it will seriously endanger human health (Raggi et al., 2018).

Surface markers of stem cells have been proposed by relevant laboratories based on biological characteristics and characterization, and different stem cell populations have been defined according to these markers (Chambers et al., 2021). These minimal positive markers that can be used to identify this stem cell population mainly include Cluster of differentiation 34 (CD34), SOX10, c-kit and Stro1, although some of them are still controversial, a relatively strict definition of stem cell characterization has been made (Mildmay-White and Khan, 2017). In addition, the expression of Sca1, Class Ⅵ intermediate filament protein (Nestin), and Glioma-associated oncogene homology 1 (Gli1) can also reflect stem cell-related properties (Calderone, 2012; Bobryshev et al., 2015; Kramann et al., 2016). However, due to the heterogeneity of stem cells and the lack of comprehensive and robust definitions of cell surface markers, there are overlapping relationships among stem cell markers.

**FIGURE 1:**
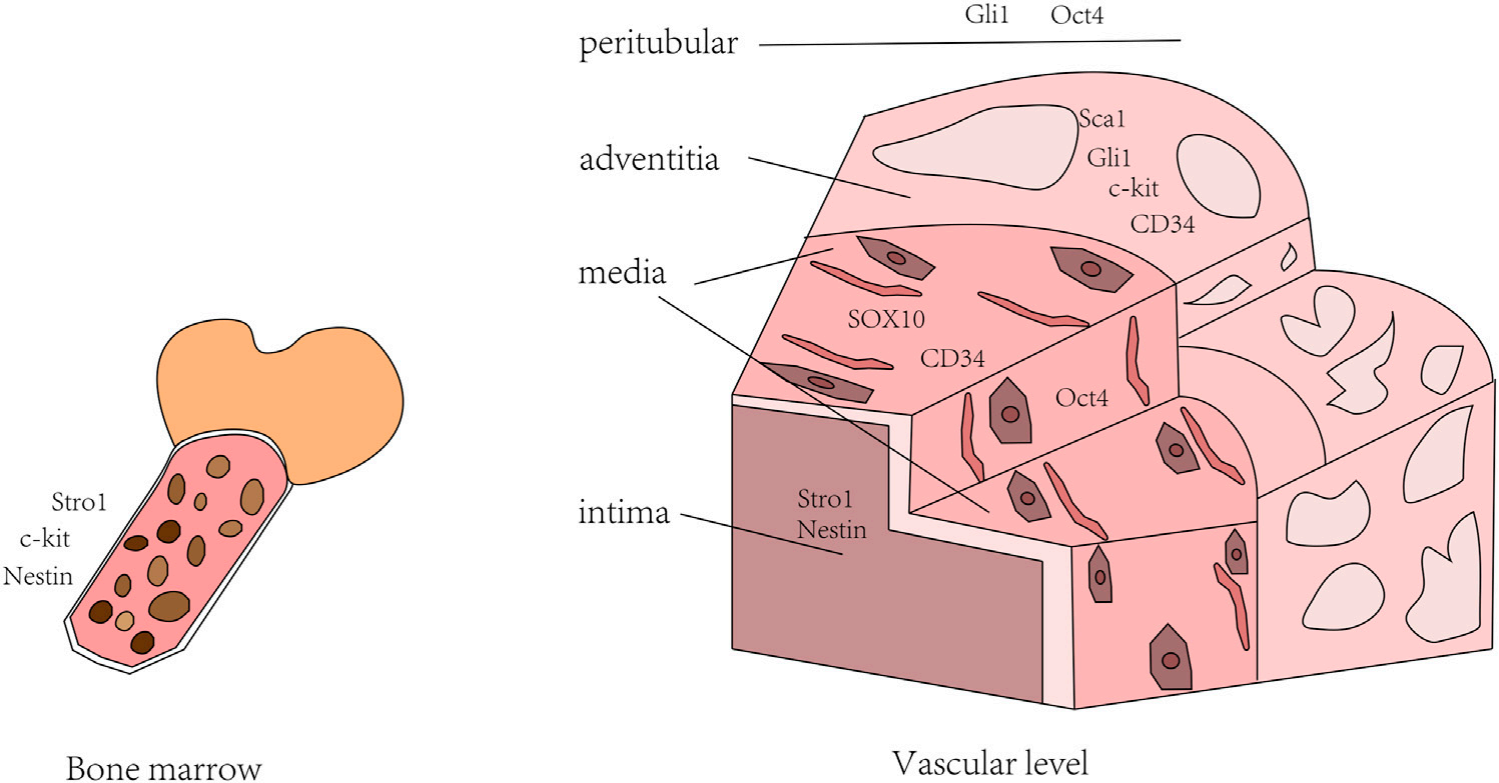
Distribution of stem cell markers in blood vessels and bone marrow.

Therefore, we could combine multiple stem cell markers to evaluate the characteristics of stem cell populations. Markers expressed by stem cells are present in all three layers of the arterial wall and bone morrow. Stem cells can specialize to form smooth muscle cells, endothelial cells, fibroblasts and osteocytes. Under certain conditions, circulating stem cells can also migrate to injured areas to participate in repair of injury and neointima formation (Jiang et al., 2021). In addition to the incomplete definition of stem cell markers, there is still no effective means to judge the process of stem cells, especially the process of differentiation into Smooth muscle cell (SMC). Thus, more studies and attempts are needed to elucidate the phenotypic differences before and after stem cell differentiation (Figure 1).

## 2 Current status and progress of atherosclerosis-related stem cells

Single-cell Ribonucleic Acid sequencing of SMCs has shown that only a modest fraction of smooth muscle cells is capable of proliferation and differentiation, and the field is moving toward an in-depth phenotypic characterization of SMCs (Liu and Gomez, 2019). In addition to medial smooth muscle, other non-smooth muscle cells, such as stem cells, endothelial cells, and derived cells, are also involved in neointima and atherogenesis of smooth muscle cells in sclerosing lesions (Wang et al., 2018). Increased stem cell proliferation accelerates clonal hematopoiesis during atherosclerosis (Heyde et al., 2021). Since the discovery of vascular stem cells, continued exploration has considerably improved our understanding of their properties and functions. Vascular stem cells are mostly quiescent but are activated in response to injury and are involved in repairing the endothelium and forming neointima (Zhang et al., 2018). In the process of SMC undergoing multiple phenotypic transitions, the expression of mesenchymal stem cell-like markers can replace cell types (Liu and Gomez, 2019). Vascular stem cells have the differentiation potential to participate in vascular remodeling, mainly by activating resident stem cells after injury, improving the ability of SMC aggregation and stem cell migration, and accelerating neointimal hyperplasia (Jiang et al., 2021). Mesenchymal stem cells also inhibit inflammatory activation of macrophages by inducing cholesterol efflux (Groenen et al., 2021). On the other hand, the important contribution of stem cell-derived exosomes in regulating stem cell differentiation, inflammation and immune response to improve atherosclerosis has been gradually recognized (Ling et al., 2021). However, due to the lack of powerful molecular markers to identify and judge stem cell differentiation processes, the cellular behavior and molecular mechanisms of vascular stem cell differentiation into SMC are still unclear. In the future, using phenotypic differences before and after stem cell differentiation to assess changes in stem cell differentiation process and cell characteristics is crucial to elucidate the behavior of vascular stem cells and the signaling pathways involved, which may provide new opportunities to promote the understanding of the functional properties of these cells and the application of clinical therapy (Jiang et al., 2021).

## 3 Sca1+ precursor cells involved in molecular phenotypic changes and signal pathways of neointima

The study of Hu et al. (2004) confirmed that there are a large number of progenitor cells that can differentiate into smooth muscle cells in the adventitia of blood vessels, and these progenitor cells are not derived from bone marrow, but participate in the formation of atherosclerosis. Sca1+ adventitial precursor cells are heterogeneous, maintain their original state in homeostasis, differentiate into a variety of effector cell types upon activation, and contribute to neointima formation and medial wall repair (Jolly et al., 2022). For a long time, it has been believed that the phenotype of smooth muscle cells in atherosclerotic lesions is different from that of medial cells, and adventitial precursor cells such as Sca1+ progenitor cells can migrate from adventitia to intima, differentiate into smooth muscle cells, and participate in atherosclerosis (Hu et al., 2004; Tang et al., 2020; Wu et al., 2021).

Endothelial cell dysfunction is known to cause endothelial inflammation as well as monocyte aggregation, promoting vascular remodeling and neointima formation (Gerhardt and Ley, 2015). However, the application of cell fate mapping and single-cell sequencing technologies has driven the study of adventitia-mediated vascular remodeling, making adventitial biology a worthwhile aspect of the field to explore (Kuwabara and Tallquist, 2017). Sca1+ progenitor cells migrate from the outer membrane to the inner membrane and participate in the formation of neointima, and are affected by multiple factors in the process. KLF transcription factor 4 (Klf4) helps maintain the Sca1 progenitor phenotype, but Klf4 has little effect on neointima formation and selectively promotes adventitial remodeling (Lu et al., 2020), ETS variant transcription factor 2 (ETV2) promotes differentiation of Sca1 progenitors into endothelial cells, however endothelial injury models suggest that Sca1 progenitors transduced with ETV2 are unable to participate in neointima formation and vascular remodeling (Le Bras et al., 2018), However, SMCs promote the migration of stem/progenitor cells from the adventitia to the neointima by releasing C-C motif chemokine ligand 2 (CCL2) and C-X-C motif chemokine ligand 1 (CXCL1) (Yu et al., 2016).

In recent years, in order to elucidate the role of Sca1+ progenitor cells in the process of vascular injury and intimal lesions, Wu et al. (2021) created a model in which Mesenchyme Homeobox 1 (Meox1) controls Sca1+ stem cells to participate in neointima formation. The study demonstrated that Meox1 promotes blood vessels through the Ras homolog family member A (RhoA)—cell division cycle 42 (Cdc42)—C-X-C motif chemokine receptor 4 (CXCR4) pathway Sca1+ progenitor cells in the adventitia migrate to the intima, and then differentiate into SMCs to participate in the formation of neointima. Tang et al. used single-cell sequencing to identify that Sca1-derived smooth muscle cells have a stronger proliferation potential than pristine smooth muscle cells after severe vascular injury. The study showed that Yap transcription factors are involved in SMC-derived expansion, so the Hippo- Yes-associated protein (Yap) pathway may be activated to participate in vascular repair (Wu et al., 2021). In addition, the integrin pathway (Xiao et al., 2007), Signal transducer and activator of transcription 3 (STAT3)- Mitogen-activated protein kinase (MAPK) pathway (Xie et al., 2017), Dickkopf wnt signaling pathway Inhibitor 3 (DKK3)/wnt family member (WNT) signaling pathway (Karamariti et al., 2018), Phosphatidylinositol 3-kinase (PI3K)/Akt serine/threonine kinase (AKT) and Nuclear factor kappa B subunit (NF-kB) signaling pathway (Wu et al., 2019) are involved in the differentiation of Sca1+ progenitor cells into smooth muscle. However, the mechanism of pathway of Sca1+ progenitor cells affecting angiogenesis and intima formation has not been widely recognized due to different methods of progenitor cell extraction and intervention measures.

For the identification of molecular phenotypes after arterial injury, they used an arterial anastomosis model and collected tissue samples after 2 weeks (short-term) and 5 weeks (longterm) of injury. The results show that Sca1-derived cells express Calponin 1 (CNN1) and Transgelin (SM22) at both stages. However, the former has not acquired a mature SMC phenotype and does not express Smooth muscle myosin heavy chain 11 (MYH11), while the latter has acquired a mature SMC phenotype and can express MYH11 (Tang et al., 2020). In the future, we can use the differences in molecular phenotypes to identify smooth muscle cells derived from Sca1+, which provides new methods and theoretical ideas for future studies. This method is also suitable for further exploring the migration mechanism of Sca1+ progenitor cells corresponding to hyperlipidemia (Kokkinopoulos et al., 2017), which can promote the discovery of therapeutic targets for vascular lesions in the future (Jolly et al., 2022), and open up new therapeutic prospects for vascular related pathological diseases.

## 4 Phenotypic changes and pathways of SOX 10+ stem/progenitor cells

Recently, more and more studies have found that the adult stem cell transcription factor SOX10 contributes to tissue encapsulation and microvascular formation (Wang et al., 2017). In the past, smooth muscle differentiation and proliferation was widely believed to play a crucial role in vascular remodeling and disease. However, recent research has shown that vascular stem cells also play an influential role. Pluripotent vascular stem cells can differentiate into neuron-like and mesenchymal stem cell-like cells, and then participate in vascular remodeling and neointima formation of smooth muscle cells. This study defines a “Vascular mesenchymal stem cell (MVSC)—Mesenchymal stem cell (MSC)—SMC” differentiation pathway, distinct from SMC dedifferentiation (Tang et al., 2012b). Since MYH11-negative cells were found to have multi-directional differentiation potential in the early stage of the experiment, studies using MYH11 as lineage tracing found that the neointima 3 weeks after injury contained proliferating cells derived from mature SMCs (Nemenoff et al., 2011), and MYH11 as a late marker of SMC (Yang et al., 2020), the switch of this phenotype after vascular injury intrigued us, Yuan et al. (2017) used lineage tracing in transgenic mice and experimented with a wire injury model and a ligation model, the results showed that SOX10+ cells may be one of the sources of heterogeneous neointima. SOX10+ vascular stem cells are quiescent under normal conditions, and arterial media only express SOX10 after vascular injury to promote proliferation and migration, thereby filling the media and creating neointima.

Yuan et al. (2017) differentiated the neointima into types Ⅰ and Ⅱ, derived from smooth muscle cells and SOX10+ cells, respectively. 2 weeks after injury, lineage tracing results indicated that Smooth muscle actin alpha 2 (ACTA2) was extremely expressed and CNN1 was lowly expressed, but MYH11 was not expressed, which is in line with the concept that vascular stem cells affect angiogenesis intimal hyperplasia after injury, and provides a more adequate theory in accordance with. Additional studies have found that macrophage-derived Matrix metallopeptidase 8 (MMP8) promotes the differentiation of SOX10+ progenitor cells into smooth muscle cells by regulating the ADAM metallopeptidase domain 10 (ADAM10)/Notch receptor 1 (Notch1) signaling pathway (Yang et al., 2020). In addition, the KIT Proto-Oncogene (KIT) pathway has also been confirmed that the neural sheath transcription factor SOX10 plays a key role in the cell survival process (Su et al., 2020).

In the future, we can use molecular phenotype-specific expression of smooth muscle cells from different sources to distinguish smooth muscle cells derived from stem cells. An in-depth study of the mechanisms and pathways associated with the differentiation of SOX10+ progenitor cells into smooth muscle cells, using this as an entry point, has the potential to provide new theoretical ideas for interventions in vascular disease and remodeling, and potentially discover new therapeutic targets.

## 5 Nestin+ progenitors involved in vascular remodeling

Nestin is an intermediate filament protein expressed in neuroepithelial stem cells or mesenchymal stem cells (Suguta et al., 2007), and as a well-known stem cell marker, it can be used as a description of stem cell-like progenitor cells in development and tissue repair (Saboor et al., 2016). Nestin was not detected in undamaged arteries, but was detected in the neointima of injured arteries (Wan et al., 2012). Nestin is also upregulated in revascularized arteries after vascular injury, especially in the neointima, suggesting that Nestin expression is involved in vascular remodeling (Oikawa et al., 2010). After further dissecting the role of Nestin in neointima, we found that Nestin is expressed in arterial neointima after balloon injury and in smooth muscle cells in human coronary lesions, representing an adaptive proliferation expression. Phenotype, and the absence of Nestin has profound effects on proliferation and migration, which together suggest that Nestin is involved in both physiological and pathological vascular remodeling processes (Tardif et al., 2015; Calderone, 2018). Although the origin of stem cells is debated, more and more studies have shown that it is certainly involved in vascular remodeling (Psaltis and Simari, 2015). On the premise that Nestin+ smooth muscle cells are derived from stem cells, we speculate that the cells may be differentiated from mesenchymal stem cells (Suguta et al., 2007; Wan et al., 2012), although there is no completely convincing research on the source of Nestin cells. Explained, but the differentiation of Nestin+ pluripotent stem cells into pericytes and smooth muscle cells is widely recognized to be involved in vascular remodeling and neovascular hyperplasia after arterial injury. Heparan sulfate variant 7 (HS7) was recently found to co-localize with Nestin, and HS7 activates AKT and MAPK transduction pathways when promoting angiogenesis and neural stem cell proliferation (Chan et al., 2020), which still needs more research support.

To obtain *in vitro* and *in vivo* expression profiles of Nestin in vascular smooth muscle cells, Oikawa et al. (2010) analyzed the localization of Nestin in vascular smooth muscle cells using the premise that 5-Bromodeoxyuridinc (Brud)+ is approximately equivalent to Nestin+. Nestin immune responses gradually decrease over time and disappear after 20 weeks of birth. Based on the properties of Nestin as a stem cell, the data suggest that Nestin+ cells maintain progenitor properties until at least 6 weeks after birth. After 2 weeks of embryos, immunohistochemistry showed that the Nestin signal coexisted with SM22 Deoxyribonucleic acid (DNA), a marker for vascular smooth muscle cells, but not fused with the signal of factor VIII-associated antigen (Oikawa et al., 2010). Saboor et al. (2016) detected the Nestin+ cells in the pulmonary artery, the smooth muscle cellspecific marker SMA positive, and the endothelial marker Platelet endothelial cell adhesion molecule 1 (CD31) negative , which is consistent with the former conclusion. In addition, this marker CNN1 was inversely correlated with Nestin expression and vascular smooth muscle proliferation in the study (Saboor et al., 2016). As a mid-to-late marker for evaluating the role of SMC in neointima, CNN1 was negatively correlated with the proliferation of vascular smooth muscle (Yang et al., 2020), suggesting that Nestin-positive cells represent a certain number of stem cells involved in vascular remodeling and neointima after arterial injury membrane formation (Yang et al., 2020). It provides a certain theoretical basis and methodology for follow-up studies of stem cells involved in atherosclerosis and neointima formation. Certain specific phenotypes of stem cell markers can also provide innovative ideas for the study and treatment of vascular diseases.

## 6 Gli1+ adventitial precursor cells with dual roles in organ fibrosis and angiogenesis

MSCs play an essential role in maintaining homeostasis and promoting repair after injury (Chen et al., 2020). Zhao et al. (2014) used lineage tracing in a mouse incisor model to find that Gli1 labeled perivascular MSC-like cells in mouse incisors, which expressed canonical MSC markers and possessed the potential for multilineage differentiation *in vitro*. Gli1+ cells are pluripotent stem cells of periodontal tissue and play a key role in periodontal tissue renewal and damage repair (Men et al., 2020).

Gli1+ MSC cells are mainly localized in pericyte niches of microvasculature and adventitia of larger vessels, and are resident cells that arise from tissue rather than circulation. Gli1+ cells have dual roles in organ fibrosis and angiogenesis (Zhao et al., 2014; Kramann et al., 2015). On the one hand, Gli1+ cells can serve as precursors of fibroblasts and differentiate into fibroblasts through proliferation after vascular injury, and Gli1+ stromal cells also play a key role in myelofibrosis (Schneider et al., 2017). Gli1+ cells retain MSC expression during fibrosis, which defines immature perivascular cells (Kramann et al., 2015). On the other hand, the periarterial Gli1+ cell portion does not express the classical MSC marker, but can generate Chondroitin sulfate proteoglycan (NG2) pericytes that express the marker (Zhao et al., 2014), and NG2 pericytes represent a subpopulation of MSCs and obtain markers of mature pericytes (Kramann et al., 2015), functions in emergency repair and intimal generation following vascular injury (Zhao et al., 2014).

Current theories suggest that the formation of atherosclerotic plaques is caused by the dedifferentiation of mature vascular smooth muscle cells after injury to differentiate into osteoblastlike cells that drive calcification of the media and intima (He et al., 2020), while dedifferentiated smooth muscle cells may derive from Gli1+ adventitial precursor cells, which is not mutually exclusive with the former model (Baker and Péault, 2016). An increasing number of studies have shown that stem/progenitor cells present in the vessel wall, such as smooth muscle precursor cells, migrate to the intima to differentiate into smooth muscle cells, and thus participate in atherosclerotic lesions and neointima formation (Baker and Péault, 2016; Kramann et al., 2016; He et al., 2020). Gli1 represents a specific marker in MSC tissue, and adventitial progenitors are thought to play a role in angiogenesis and disease development (Psaltis and Simari, 2015).

Through lineage tracing of Gli1+ vascular cells, Kramann et al. (2016) showed that adventitial cells migrate into the capsular-media and neointima, and subsequently differentiate into smooth muscle cell-like outlines, which in turn differentiate into osteoblasts, which drive the process of vascular calcification. Using a well-established model of femoral artery wire injury (Sata et al., 2000), they demonstrated through inducible genetic fate tracing that Gli1+ progenitors are an important source of adventitial cells for vascular smooth muscle cells and contribute to arterial wall repair after acute injury (Kramann et al., 2016), mainly reflected in the Gli1+MSC cells involved in the formation of neointima (Kramann et al., 2016; Song et al., 2020). Gli1+ cells obtained the expression of smooth muscle cell marker α-SMA (ACTA2), smooth muscle protein and calponin in the middle and late stages of isolation or under the condition of adding Platelet-derived growth factorbb (PDGF-BB) and Transforming growth factor beta (TGF-β). In contrast, none of these markers could be detected (Kramann et al., 2016). Furthermore, single-cell transcriptional analysis showed that numerous Gli1+ adventitial precursor cells naturally express Transgelin (SM22) (Baker and Péault, 2016; Kramann et al., 2016). Studies have shown that induction of the Hedgehog/Klf4 pathway is the basis for the reprogramming of smooth muscle cell-derived stem progenitor cells (Lu et al., 2020). We speculate that Gli1+ progenitor cells are also present in human arteries and that molecular phenotype-specific expression of Gli1+ cells provide a new theoretical avenue for followup studies, but whether they can be used as progenitor mesenchymal stem cells and for vascular calcification remains open. Treatment targets still need to be studied further.

## 7 Different roles of c-kit+ stem/progenitor cells derived from vascular wall and bone marrow

The crucial role of smooth muscle cells in the progression of atherosclerotic lesions is universally recognized (Bennett et al., 2016), but the origin of smooth muscle cells in the neointima remains controversial. In the past, it was thought that the medial smooth muscle cells dedifferentiate and then migrate to the intima to proliferate to form the neointima (Ross, 1999), and more and more studies have shown that there are a large number of progenitor cells in the adventitia of atherosclerotic lesions, and these progenitor cells can It expresses markers such as c-kit, Sca1, and CD34, and is involved in neointima formation and atherosclerotic lesions, and may be the source of endothelial cells and smooth muscle cells (Torsney et al., 2007). In addition to medial smooth muscle cells, bone marrowderived smooth muscle precursor cells as well as vascular-resident stem/progenitor cells may be involved in this process (Takamiya et al., 2006; Wang et al., 2007; Skartsis et al., 2014; Chen et al., 2018; Ni et al., 2019).

Endogenous c-kit+ stem/progenitor cells have been reported to play a role in tissue repair by differentiating into endothelial cells and smooth muscle cells during the repair of injured arteries (Ellison et al., 2013). Blockade of c-kit attenuates neointima formation (Skartsis et al., 2014; Chen et al., 2018), conversely demonstrating an important role for c-kit in intima formation. Chen et al. (2018) established a vascular injury model and used genetic lineage tracing to find that c-kit+ stem cells mainly differentiate into monocytes/macrophages and granulocytes during intima formation, which promote inflammatory aggregation after injury, thereby infiltrating neointimal lesions. Interestingly, they suggest that the potential of c-kit+ cells to differentiate into endothelial cells and smooth muscle cells is low during intima formation, and that it is mainly through intima infiltration and differentiation that lesion formation is facilitated. Although c-kit+ cells are present in both the vessel wall and bone marrow, data demonstrate that bone marrow-derived c-kit+ cells are the main source of neointima formation (Chen et al., 2018), Progenitors cells expressing the c-kit marker are recruited to the vessel wall and differentiate mainly into monocytes/macrophages and granulocytes, rarely into endothelial and smooth muscle cells, causing vascular inflammation to promote remodeling after injury (Chen et al., 2018). Vascular inflammation plays a key role in the process of neointimal hyperplasia and vascular remodeling (Libby, 2021). On the other hand, cytokine mobilization of c-kit+ stem cells to promote re-endothelialization after angioplasty is a feasible strategy (Takamiya et al., 2006). In a mouse arterial transplantation model, smooth muscle cells in the neointima are populated with c-kit+ cells derived from non-bone marrow, and bone marrowderived c-kit+ cells can produce leukocytes, which together promote neointima formation (Ni et al., 2019). This does not conflict with Chen et al. exclusively. The former study in a mouse allograft model showed that c-kit+ cells can act as a major contributor to the accumulation of neointimal smooth muscle cells, and bone marrow-derived c-kit+ cells may also be an influential factor in neointima formation (Ni et al., 2019). The latter believes that endothelial cells and smooth muscle cells are fewer generated mainly in the natural pathophysiological process of the neointima, and cannot be the main source of endothelial repair and smooth muscle accumulation after vascular injury (Chen et al., 2018). Differences in results may be related to the vascular disease model employed and the severity of vascular injury.

Ni et al. (2019) found that Stem cell factor (SCF)/c-kit axis migration can activate the downstream MAPK and Jun Proto-Oncogene (JUK) pathways to induce differentiation into smooth muscle cells, and glucose metabolism also plays a key role in the induction of differentiation. Previous related studies have shown that c-kit precursor cells promote angiogenesis in tissue grafts by regulating the Wnt/Klf4 pathway (Campagnolo et al., 2015). These findings provide current insights into the mechanisms of neointima, and these studies of c-kit+ cells provide theoretical foundations and potential targets for subsequent stem cell therapies for vascular diseases.

## 8 Contribution of Stro-1+ mesenchymal progenitor cells to myofibroblast phenotype after vascular injury

Stro-1 is a cell membrane one-way protein that is translocated from the endoplasmic reticulum to the cell membrane in the presence of intracellular calcium depletion (Barkhordarian et al., 2011). As one of the most famous markers of bone marrow mesenchymal stem cells, Stro-1 plays a unique role in the research of bone marrow mesenchymal stem cells (Lin et al., 2011). Immunofluorescence staining showed that Stro-1 was present in nerve fibers, vascular smooth muscle cells, pericytes, and endothelial cells (Yoshiba et al., 2012). Stro-1+ cells are mesenchymal stem cells with multi-lineage differentiation potential, including chondrocyte differentiation, adipocyte differentiation, smooth muscle cells (Kobayashi et al., 2004), which can participate in the repair process after tissue damage. Progenitors expressing Stro-1 are recruited in the perivascular setting and maintain the inflammatory response by enhancing chemotaxis to neutrophils and monocytes/macrophages (Ward, 2010). Stro-1 antigen, as a typical surface antigen of bone marrow stem cells, has inflammatory/immune effects during arterial disease (Ryer et al., 2015). Stro-1 + stem cells are induced to migrate selectively, while mesenchymal stem cells and pericytes have strong osteogenic potential (Doherty et al., 1998; Dennis et al., 2002; Chmilewsky et al., 2013), increased differentiation of osteoblasts may promote tissue repair and vascular remodeling (I et al., 2011). Studies of arterial intima in patients with pulmonary hypertension have shown that the Stro-1+ stem cell population has the ability to differentiate into adipocytes and osteoblasts, thus contributing to vascular lesions (Firth et al., 2010). Dental pulpderived mesenchymal stem cells generate reparative odontoblast-like cells (Yoshiba et al., 2012), and injection of a mixture of hyaluronic acid, butyric acid and retinoic acid can lead to the recruitment of Stro-1+ stem cells and enhance MSC-mediated cytotoxicity *in vivo* (Lionetti et al., 2010). In vascular lesions, the myofibroblast phenotype predominates in endarterectomy tissue (Firth et al., 2010), and meaningfully, mechanical stress has been shown to promote the expression of SMC-like properties of bone marrow stromal cells (Kobayashi et al., 2004). Immunofluorescence and blotting indicated that both α-SMA were expressed, while the proportion of SM-MHC increased with the prolongation of culture time, and was mainly expressed in the later stage of cell differentiation (Kobayashi et al., 2004). The neointima is derived from mesenchymal progenitor cells of the bone marrow, which in turn repair vascular damage (Kobayashi et al., 2004). However, bone marrow-derived cells are not the only players involved in vascular repair and homeostasis, and cells with morphological and immunophenotypic properties of mesenchymal stem cells are also present in human elastic and muscular arteries (Pasquinelli et al., 2010). With regard to the proliferation and osteogenic differentiation of periodontal ligament and dental pulp stem cells, studies have demonstrated that MAPK pathway and Wnt/β-catenin signaling pathway (Torsney and Xu, 2011) play an indispensable role and are closely related to the differentiation of mesenchymal cells into smooth muscle. The association of cellular processes requires additional study.

By detecting the expression of Stro-1, it was preliminarily identified that bone marrow stromal precursor cells can form fibroblasts (Firth et al., 2010), which in turn proved the contribution of bone marrow-derived circulating progenitor cells. At the same time, mechanical stress can promote the expression of smooth muscle-like properties, which reflects the effect of vascular pressure on the neointima (Kobayashi et al., 2004), and high glucose can induce endothelial cell-mesenchymal transformation into chondrocyte-like cells, which are then involved in the calcification of the vascular media (Tang et al., 2012a). Both influences are involved in the progression of neoplasia and atherosclerosis through the pathway of Stro-1+ mesenchymal cells. It provides us with a current strategy to understand the pathogenesis of vascular disease and the prevention of arteriosclerosis.

## 9 Limited contribution of CD34^+^ resident stem cells to new smooth muscle cells

Stem/progenitor cells may be involved in vascular repair and intima formation processes. Vascular progenitor cells including endothelial progenitor cells and CD34^+^ cells migrate to the intima during vascular injury and differentiate into vascular smooth muscle cells (Torsney and Xu, 2011). The presence of angiogenic mesenchymal cells in the human thoracic aorta, including both CD34 and ckit cell populations, is associated with proliferative markers (Pasquinelli et al., 2007). In the study of the great saphenous vein, it was found that CD34+/CD31-cells have the potential to differentiate into pericytes (Campagnolo et al., 2010), and pericytes can already differentiate into adipocytes to form lipid nuclei, thereby promoting the formation of plaques in atherosclerotic lesions (Canfield et al., 2000). In a mouse vascular graft model, Tasi suggested that the neointima is heterogeneous, expressing stem cell markers including Sca1, c-kit, CD34, and that the smooth muscle cells in the plaques are derived from the vessel wall rather than myeloid cells (Tsai et al., 2012). However, the contribution of the CD34^+^ resident stem cells of the progenitor cells to the new smooth muscle cells is limited. CD34^+^ stem cells isolated from adventitia can differentiate into smooth muscle cells *in vitro* and express the early marker SM22 and rarely the late marker SM-MHC, but the vessels are damaged. Rather than participating in intima formation, smooth muscle cells are produced in small numbers and only migrate to the media and maintain equilibrium (Shen et al., 2016). At the same time, it has been shown in stem cell targeted therapy that bone marrow mesenchymal stem cells can replace damaged cells and promote neointima formation in atherosclerosis. This effect is worthy of recognition (Hashem et al., 2021).

The existence and localization of CD34^+^ stem cells in the blood vessel wall are relatively clear, but there are still differences in the formation of atherosclerotic plaques and neointima by adventitial CD34^+^ resident stem cells, which may be related to the selection of arterial and venous sites or the experimental method adopted. Related to differences in animal models, studies have found that CD34^+^ stem cell-derived SMCs are mediated by Ras Homolog Family Member A (RhoA) and Ca^2+^/CaM/Myosin Light Chain Kinase (MLCK)-dependent pathways (Vazão et al., 2011), and more studies are needed to explore this mechanism. However, the efficacy of bone marrow mesenchymal stem cells on neointima deserves recognition and offers excellent prospects for the prevention and treatment of vascular diseases.

## 10 Activation of stem cell transcription factor Oct4 regulates phenotypic shift

Octamer-binding transcription factor 4 (Oct4) was first discovered in early embryos and germ cells, and as an animal transcription factor, it determines the formation of pluripotent stem cells (Nichols et al., 1998). Generally, Oct4 expression is restricted to pluripotent stem cells, and recent studies have found that under pathological conditions, Oct4 activation in a variety of tumors (Gkountela et al., 2019) and atherosclerotic lesions (Cherepanova et al., 2016; Alencar et al., 2020) is associated with migration of cell types (Ding et al., 2021). During the development of atherosclerosis, Oct4 is activated as a key transcription factor that maintains stem cell dedifferentiation and plays an important role in the regulation of the phenotypic transition of vascular smooth muscle cells (Cherepanova et al., 2016), but this evidence is controversial. After using smooth muscle cell-specific knockdown of Oct4, Cherepanova found a decrease in plaque stability, which may be related to the reduced number of SMCs within the lesions due to impaired SMC migration (Cherepanova et al., 2016). In view of the regulation of stem cell pluripotency genes on the pathogenesis of advanced atherosclerotic lesions, Alencar believes that activation of Oct4 may play a beneficial role in the stabilization of atherosclerotic plaques, which is consistent with the former view, and found that Klf4 and Oct4 showed virtually opposite genome-wide influence regulation on the arteriosclerosis protective phenotype (Alencar et al., 2020). Activation of Oct4 in vascular smooth muscle has a protective effect on arteriosclerosis.

However, arterial injury-induced neointima formation and diet-induced atherosclerosis differ in pathological and mechanistic as well as genetic factors (Ding et al., 2021), knockout of Oct4 significantly impairs perivascular cell migration and promotes Vascular leakage, which in turn inhibits angiogenesis (Hess et al., 2019). Activation of the stem cell factor Oct4 is involved in the remodeling of the vascular network after vascular injury (Hess et al., 2019). The level of Oct4 in the arterial intima is significantly increased after carotid artery injury (Ding et al., 2021), which in turn regulates the formation of neointima. The expression and localization of Oct4 mainly depends on the co-localization of *a*-SMA in the endocardium and proliferating cell nuclear antigen (PCNA) in the nucleus of vascular smooth muscle cells. Clear *in vitro* experiments also proved that the overexpression of Oct4 promotes the proliferation and migration of smooth muscle cells (Yang et al., 2020). Studies have also shown that Oct4 promotes the generation of smooth muscle cells from pluripotent stem cells and regulates the transcriptional activation of SM22 through the DDK3/Wnt signaling pathway (Karamariti et al., 2013), thereby promoting the repair and regeneration of blood vessels.

Despite epigenetic silencing during gastrulation (Hess et al., 2019), the stem cell factor Oct4 can be activated under specific conditions to play a protective role during atherosclerotic lesions and angiogenesis. Given that Oct4 can play an important role in the process of stem/progenitor cell differentiation, it is seldom expressed in somatic cells and has fewer potential side effects, and may become a new target for vascular therapy in the future (Ding et al., 2021). However, the mechanisms and factors responsible for Oct4 activation in somatic cells and the SMC phenotypic transition need to be further investigated in order to identify therapeutic targets (Table 1).

**TABLE 1:** Stem/Progenitor cells differentiation function and phenotype.

Stem cell markers	Stem/progenitor cells	Stem cell location	Function	Differentiation potential	Phenotype	References
Sca1	Sca1+ precursor cells	adventitia	Migrate to intima, media, SMC accumulation and differentiation Promotes the generation of circulating SMC precursor cells Acts on the epithelium and promotes mesenchymal transition	SMC Endothelial cell	SM22 (+) CNN1 (+) MYH11 (−)	Tang et al. (2020)
SOX10	SOX10+ pluripotent vascular stem cells	Medial membrane	Differentiation into SMC and chondrocytes after injury Vascular remodeling, intimal hyperplasia	SMC Chondrocyte	ACTA2 (+) CNN1 (+) MYH11 (−)	Yuan et al. (2017)
Nestin	Nestin+ mesenchymal stem cells	inside of blood vessels arterial media marrow	Vascular re-endothelial Bone marrow mesenchymal stem cells participate in vascular remodeling myofibroblasts	SMC Endothelial cell Myofibroblast	ACTA2 (+) CD44 (+) CNN1 (−) CD31 (−)	Oika wa et al. (2010), Saboor et al. (2016), Yang et al. (2020)
Gli1	Gli1+ MSC-like precursor cells	pericyte niche adventitia	Vascular endothelial cell generation. Formation of myofibroblasts, vascular fibrosis Neointima, formation of osteoblast-like cells, calcification of blood vessels	Endothelial cell Myofibroblast Osteoid cell	ACTA2 (+) CNN1(−)	Kramann et al. (2016)
c-kit	c-kit+ stem/progenitor cells	marrow Vascular wall in the adventitia, intimal lesions	Bone marrow-derived cells differentiate into myeloid cells that promote immune inflammation, improve lesions, and promote myelodysplasia. The source of the vessel wall is differentiated into EC and SMC, which act on the damaged vessel intima	Myeloid cell SMC Endothelial cell	ACTA2 (+) MYH11 (−)	Chen et al. (2018)
Stro-1	Stro-1+ mesenchymal stem cells	marrow small arterial endothelial cells adipocyte endothelial cells	Homing and angiogenesis Chondrocyte differentiation, adipocyte differentiation, smooth muscle cells	Chondrocyte Adipocyte SMC	ACTA2(+) SM22 (+) SMTN (−) MYH11 (−)	Koba yashi et al. (2004), Yoshiba et al. (2012)
CD34	CD34^+^ stem/progenitor cells	Vascular media and adventitia Endothelial cells	Migrating to the outer layers of the media and maintaining the homeostasis of the media Differentiating into pericytes, adipocytes, chondrocytes, osteoblasts, and promoting plaque formation	Pericytes, Adipocytes, Chondrocytes, Osteoblasts	SM22 (+) CD31 (−) MYH11 (−)	Shen et al. (2016)
Oct4	Oct4+ stem cell pluripotency factor	perivascular cells Vascular media and lesions marrow	Promotes bone marrow stem cell homeostasis and angiogenesis. Promoting SMC migration and stabilizing patches Migration and recruitment of perivascular cells promotes SMC proliferation and stabilizes new blood vessels	Perivascular cell SMC	ACTA2 (−) CNN1 (−) MYH11 (−) PCNA (+)	Yang et al. (2020), Karamariti et al. (2013), Ding et al. (2021)

ACTA2 = α-SMA, Actin alpha 2 smooth muscle; CNN1, Calponin 1; MYH11 = SM-MHC, Myosin heavy chain 11; SM22 = TAGLN, transgelin; PCNA, proliferating cell nuclear antigen; SMTN, smoothelin; CD31, Platelet endothelial cell adhesion molecule; CD44, Transmembrane adhesion glycoprotein.

## 11 Stem cell differentiation promotes pathways associated with changes in SMC phenotype

Summarizing the roles of the above-mentioned various stem/progenitor cells in the differentiation process of phenotypic changes, we found that in stem/progenitor cells positive for a variety of stem cell markers, the Wnt/β-catenin pathway plays an essential role in promoting SMC proliferation in stem cells. It plays an influential role in the process of endothelial cell fibrosis and reendothelialization, activation of Wnt signaling pathway can induce differentiation of stem cells and reprogramming of SMC (Karamariti et al., 2013; Campagnolo et al., 2015; Lu et al., 2020; Yang et al., 2020), mainly including two signal activation pathways, Dickkopf3 and Hedgehog. Channel activation favors adventitial remodeling and fibrosis, and promotes SMC proliferation and reendothelial in the neointima (Campagnolo et al., 2015). After being stimulated by vascular injury, the secretion of Vascular Endothelial Growth Factor (VEGF) and TGF-β stimulates the upregulation of the MAPA pathway, the phosphorylation of JUN, and the elevated expression of osteogenic genes in Stro-1 stem cells (Li et al., 2021). SMC proliferation after phenotype conversion of Nestin+ stem cells (Chan et al., 2020), Thereby promoting angiogenesis and osteogenic differentiation, these conclusions need additional research support. Activation of the RhoA/Rho kinase signaling pathway can regulate actin polymerization and MLCK phosphorylation (Vazão et al., 2011; Ni et al., 2019), improve contractility and viability at the cellular level, and promote SMC proliferation and angiogenesis. In addition, some signaling pathways are activated in specific stem cell markers, PI3K/Akt signaling pathway promotes SMC differentiation dependent on SDF-1α expression (Wu et al., 2019), Rac Family Small GTPase 1 (Rac1)/Cdc pathway can participate in intimal formation as a co-control Sca1+ stem cell (Ni et al., 2019), Ca^2+^/CaM signaling pathway mediates cell contraction of stem cells (Vazão et al., 2011). To date, there has been insufficient research on the activation of signaling pathways during stem cell differentiation after vascular injury, and additional theoretical support is needed. Due to the complex biological properties of SMCs, Huize Pan conducted a singlecell analysis of the process of SMC phenotype changes in atherosclerosis, revealing that SMC-derived intermediate cells have the characteristics of differentiation into macrophages and fibrosis, prompting the expression of The pathways regulated in the process of type switching mainly include NF-κB signaling pathway, PI3K/Akt signaling pathway, G protein-coupled receptor (GPCR) pathway, Hedgehog/Notch/Wnt signaling pathway, which is consistent with the above conclusions in the stem cell field in recent years on differentiation process pathways. If there is an intersection, these pathways may be activated in both differentiation modes, and there may be differences in the regulation of the differentiation process after activation, or it may be caused by the overlap of the downstream pathways in the two differentiation modes, which needs to be explored in-depth (Table 2).

**TABLE 2:** Signaling pathways in different stem cell populations: similarities and contributions.

Signaling pathways	Stem cell marker	Functional action	Differentiation potential
PI3K/AKt	Sca1, Gli1	1. Dependent regulation of SDF-1α expression promotes differentiation of vascular smooth muscle cells (Wu et al., 2019)	SMC Myofibo blast
		2. Phosphorylation of AKT and SMAD^2^/3 to promote the expression of myofibroblast markers (Song et al., 2020)	
Wnt/DKK3/Hedgehog	Sca1 Gli1, c-kit, Oct4, Stro-1	1. DKK 3 induces Sca 1 + vascular progenitor cells and fibroblasts to differentiate into SMC by activating TGF-β/ATF 6 and Wnt signaling pathways (Karamariti et al., 2018)	SMC
		2. Hedgehog/WNT/β-catenin/Klf4 signaling pathway regulates SMC reprogramming and progenitor phenotype, promoting spontaneous adventitia remodeling (Lu et al., 2020)	
		3. Regulation of Wnt/Klf 4 Pathway Promotes Angiogenesis in Tissue Engineering Grafts (Campagnolo et al., 2015)	
		4. DKK 3 signal pathway promotes stem cells to differentiate into SMC, forming tissue engineering blood vessel (Karamariti et al., 2013)	
MAPA-related	Stro-1, c-kit, Nestin, Sca1	1. Activation of MAPK Pathway by TGF-β1 Promotes hDPSC Viability and Osteogenic and Odontogenic Differentiation (Li et al., 2021)	Osteoblast SMC
		2. It promotes cell migration and downstream activation of small GTPases through the SCF/c-kit axis, MEK/ERK/MLC signaling pathway. TGF-β1 induces differentiation of c-Kit cells into SMCs *via* HK (hexokinase)-1-dependent metabolic reprogramming (Ni et al., 2019)	
		3. Promotes angiogenesis *via* VEGF receptors (Chan et al., 2020)	
		4. Leptin receptor and transcriptional activator 3-Rac1/Cdc42-ERK-FAK pathway promote Sca-1 progenitor cell migration and neointimal formation (Xie et al., 2017)	
RhoA/Rac1/cdc42	CD34, kit, Sca1	1. Activation of Rho/Rho Kinase-dependent Pathway Mediates Contraction (Vazão et al., 2011)	SMC
		2. RhoA/Rac1/cdc42 may act as a downstream pathway of signal transduction (Ni et al., 2019)	
		3. Control of Sca-1-positive stem cells in neointimal formation by the synergistic effect of Rho/CDC 42 and SDF-1α/CXCR 4 (Wu et al., 2021)	
RAGE/NFκB	Sca1	Direct activation of RAGE and NF-κB induces SDF-1α to maintain intermediate state of stem cells and promote neointimal formation in injured arteries (Wu et al., 2019)	Endothelial cell
Ca^2+^/CaM	CD34	Activation of Ca^2+^/CaM/MLCK Pathway Mediates Stem Cell Contraction (Vazão et al., 2011)	Pericytes
ADAM10/Notch1	Sox10	1. Signaling Axis Activates SMC Differentiation and Phenotypic Change of AdSPCs (Yang et al., 2020)	SMC
		2. Regulation of VSMC proliferation and neointimal formation by β-catenin (Xiao et al., 2014)	
Ras	Nestin	Induction of Nestin Expression by Raf and ERK Phosphorylation Promotes Phenotypic Switching (Klein et al., 2014)	SMC Endothelial cell
Hippo	Sca1	After activation, Yap regulates vascular repair and regeneration and arterial wall thickness (Tang et al., 2020)	SMC

### 11.1 Clinical significance

As our understanding of the importance and mechanisms of stem/progenitor cells involved in vascular repair and neointima formation has grown, two approaches have emerged for therapeutic approaches: on the one hand, influencing the behavior of endogenous vascular stem cells to intervene in disease development; on the other hand, infusion of exogenous stem cells can promote disease reversal and recovery from vascular injury. An increasing number of immature stem cells with differentiation potential, such as mesenchymal stem cells and pluripotent stem cells, have been reported in cardiovascular disease and peripheral arterial disease (Gorecka et al., 2019; Yamanaka, 2020).

Since atherosclerosis is an age-related disease and vascular function continues to be impaired with age (Maruhashi et al., 2020). However, the relationship between age-related vascular changes and stem cell loss in tissues is poorly understood (Chen et al., 2021). Progressive progenitor cell loss may lead to the development of atherosclerosis (Rauscher et al., 2003). The morphological features of senile vascular aging are fibrous cap formation, arterial calcification and elastic lamina fragmentation (Zhang et al., 2020). In-depth analysis of senescent tissues has confirmed that vascular depletion is a major marker of senescence and that loss of vascular abundance in pericytes impairs fibrotic differentiation (Chen et al., 2021). The process of vascular aging is also closely related to the biology of impaired stem cells, and further studies of cellular heterogeneity in vascular aging are warranted (Ungvari et al., 2018). Aging affects the activity of stem cells and weakens their ability (Saçma et al., 2019). Senescent cells induce a pro-inflammatory phenotype in blood vessels (Mazini et al., 2020; Parvizi et al., 2021). It may impair the function of circulating progenitor cells by promoting cell proliferation and migration, and/or enhancing inflammation and oxidative stress (Heiss et al., 2005). Vascular cellular and molecular aging processes also affect the venous and lymphatic systems, causing a variety of disease physiology (Ungvari et al., 2018). There is a potential link between age-related lymphoid dysfunction and amyloid pathology (Kress et al., 2014). Senescent cells signal through secreted factors to reduce stem cell regeneration and accelerate vascular aging (Donato et al., 2018). Stem cell depletion is an important mechanism of vascular aging (López-Otín et al., 2013). Supplementing stem cells can not only induce cell rejuvenation and accelerate cell repair and regeneration (Mazini et al., 2020), and can also enhance the dryness and anti-aging properties of resting cells (Baker, 2007). However, poor post-transplant survival, poor targeting and ethical issues still limit clinical use (Fennema et al., 2018; Poulos, 2018).

The availability of stem cells that are positive for the stem cell markers we discussed has increased significantly in recent years, but clinical trials and therapies using these vascular and bone marrow stem cells are still lacking. So far, human trials examining the use of vascular stem cells in vascular disease have not been widely recognized. For atherosclerosis and related diseases, most clinical trials have focused on disease treatment using bone marrow mesenchymal stem cells and endothelial progenitor cells. Studies on the underlying mechanisms and pathways of stem cells affecting angiogenesis and neointima formation show that stem cells play a greater role in disease treatment (Karamariti et al., 2018; Wu et al., 2019; Yang et al., 2020). Although our focus here is on macrovascular plaque-related diseases, bone marrow mesenchymal stem cells and endothelial progenitor cells have also been used for angiogenesis in microvascular disease (Kaushik and Das, 2019; Cooke and Meng, 2020).

The purpose of this review is to summarize the molecular markers that can be used to locate stem cells in the vascular wall, and to judge the differentiation process of stem cells by using the molecular phenotype changes before and after the stem cells participate in intima formation, so as to facilitate our research on the molecular mechanism and signal pathway of stem cells participating in intima formation and vascular repair. However, the coverage of stem cell markers in this review is still not comprehensive, and the definition of stem cells by some markers is controversial. It has also been theorized that the current definition of stem cell surface markers is not robust enough and that a combination of stem cell markers is needed to assess stem cell properties. In this review, molecular phenotypic changes are focused on stem cell differentiation processes into SMC, endothelial cells and fibroblasts. The selection of molecular phenotypes is still relatively limited, and some phenotypes have opposite conclusions in different studies. In the future, more specific phenotypes can be sought to define the differentiation process. The data for stem cell marker studies is basically from animal models. It is unclear whether the results obtained can be applied to human diseases due to the large differences in vascular structure and pathophysiology. Since this review is a summary analysis of the stem cell differentiation signaling pathway, only the overall direction and function of the pathway is summarized, which provides a reliable research idea for subsequent mechanistic studies of stem cells.

### 11.2 Summary and perspectives

Extensive research data indicates that various stem/progenitor cells are involved in angiogenesis and disease, including the formation of atherosclerotic neointima. Due to the uneven distribution of stem cells in different regions of the vessel wall, the susceptibility of different vessel stages to disease is also different (Kayashima and Maeda-Smithies, 2020). During neointima formation and thickening, smooth muscle cells, endothelial cells, inflammatory cells and stem cells present in blood vessels may interact and influence each other. Recent studies have shown that identification of vascular stem cell markers and alternative techniques for identifying suitable cellular targets and understanding their underlying regulatory mechanisms can lead to the development of effective treatments for vascular diseases. To date, some progress has been made in this area, but several issues still need to be addressed. First, stem cells are heterogeneous and diverse, and it is necessary to confirm whether the objects between different groups of studies are the same population and whether there is a clear relationship between the proliferation and synthesis of smooth muscle cells. It is critical to distinguish between different stem cell populations by exploiting differences in expression profiles of specific markers between different populations. Second, vascular stem cells modify their quiescent and activated states during angiogenesis and repair after *in vivo* vascular injury. They are affected by microscopic factors *in vivo* and further work is needed to identify niches of vascular stem cells. Third, vascular stem cells may proliferate and differentiate rapidly during the early stages of neointima formation, which is difficult to capture directly by immunohistology. To this end, we can try to address this issue using techniques from genetic lineage tracing. Alternatively, we can indirectly determine stem cell processes by distinguishing molecular phenotypes between smooth muscle cells differentiated from stem cells and smooth muscle cells derived from de-differentiated pristine smooth muscle. Fourth, it is precisely because the activation and differentiation of stem cells are regulated by various microscopic factors such as physiology and pathology, we need to clarify the behavior of vascular stem cells under pathological conditions under single and multiple factors, and understand the underlying mechanism of stem cell behavior, inspired by the successful treatment of atherosclerotic diseases in recent years, to create a more targeted treatment method with fewer side effects. Fifth, the acquisition of vascular wall stem cells is becoming increasingly feasible, so cell therapy holds great promise for disease treatment. While stem cell transplantation has been shown to be safe and beneficial for tissue regeneration, the mechanisms by which it works are not thoroughly understood. Because phenomena in humans are different from those in animals, ethically scrutinized clinical trials remain valuable to improve our understanding of the underlying mechanisms and to lay the necessary theoretical foundation for the design of future studies. Finally, beyond the delivery of exogenous stem cells for therapeutic purposes, the recruitment of endogenous stem cells or potential therapeutic targets for stem cells requires more investigation. Learning to use current technological tools such as vascular tissue culture and transgenic animal models can accelerate progress in vascular stem cell biology research, and in the future, additional diagnostic and therapeutic measures can be developed to prevent and treat vascular diseases.

## Glossary

Sca1: Stem cell antigen-1
SOX10: Sry-box transcription factor 10
Stro-1: Stromal cell antigen 1
c-kit: Stem cell growth factor receptor kit
CD34: Cluster of differentiation 34
Nestin: Class Ⅵ intermediate filament protein
Gli1: Glioma-associated oncogene homology 1
SMC: Smooth muscle cell
Klf4: Klf transcription factor 4
ETV2: Ets variant transcription factor 2
CCL2: C-C motif chemokine ligand 2
CXCL1: C-X-C motif chemokine ligand 1
Meox1: Mesenchyme homeobox 1
RhoA: Ras homolog family member A
Cdc42: Cell division cycle 42
CXCR4: C-X-C motif chemokine receptor 4
Yap: Yes-associated protein
STAT3: Signal transducer and activator of transcription 3
MAPK: Mitogen-activated protein kinase
DKK3: Dickkopf wnt signaling pathway Inhibitor3
WNT: Wnt family member
PI3K: Phosphatidylinositol 3-kinase
AKT: Akt serine/threonine kinase
NF-kB: Nuclear factor kappa B subunit
SM22 = TAGLN: Transgelin
MYH11 = SM-MHC: Smooth muscle myosin heavy chain 11
MVSC: Vascular mesenchymal stem cell
MSC: Mesenchymal stem cell
ACTA2 = α-SMA: Smooth muscle actin alpha 2
MMP8: Matrix Metallopeptidase 8
ADAM10: Adam metallopeptidase domain 10
Notch1: Notch receptor 1
KIT: KIT Proto-Oncogene
Brud: 5-Bromodeoxyuridinc
HS7: Heparan sulfate variant 7
DNA: Deoxyribonucleic acid
CD31: Platelet endothelial cell adhesion molecule 1
NG2: Chondroitin sulfate proteoglycan
PDGF-BB: Platelet-derived growth factor-bb
TGF-β: Transforming growth factor beta
PDGF: Platelet-derived growth factor
SCF: Stem cell factor
JUN: Jun Proto-Oncogene
RhoA: Ras Homolog Family Member A
MLCK: Myosin Light Chain Kinase
OCT4: Octamer-binding transcription factor 4
PCNA: Proliferating cell nuclear antigen
VEGF: Vascular Endothelial Growth Factor
PI3K: Phosphoinositide-3-kinase
Rac1: Rac family small GTPase 1
SMTN: Smoothelin
CPCR: G protein-coupled receptor
CD44: Transmembrane adhesion glycoprotein.
